# The rare entity of bilateral and unilateral neuroendocrine metastases to the breast: a case series and literature review

**DOI:** 10.3332/ecancer.2020.1123

**Published:** 2020-10-15

**Authors:** Paola Zagami, Eleni Kandaraki, Giuseppe Renne, Franco Grimaldi, Francesca Spada, Alice Laffi, Nicola Fazio

**Affiliations:** 1 Division of Gastrointestinal Medical Oncology and Neuroendocrine Tumours, European Institute of Oncology, IEO, IRCCS, Milan 20132, Italy; 2 Department of Pathology, European Institute of Oncology, Milan 20132, Italy; 3 Endocrinology and Metabolism Unit, University of Udine, Italy

**Keywords:** bilateral, breast metastases, neuroendocrine, neuroendocrine neoplasms, neuroendocrine tumours

## Abstract

**Introduction:**

Primary neuroendocrine neoplasms (NENs) in the breast are very rare. Until 2011, the prevalence was 0.1% of all breast lesions and 1% of all NENs, whereas metastatic breast NENs represent 1%–2% of all breast tumours. However, it seems that over the last 5 years the diagnostic frequency of breast NENs has increased, probably for more alert specialists and advanced diagnostic tools, leading to a prevalence of 2%–5% of diagnosed breast cancers, mostly in the elderly population. Breast metastases from extramammary malignancies are uncommon and bilateral ones are even more uncommon, with few reported in the literature. We describe four clinical settings of breast metastases from different NENs and the multidisciplinary approach for diagnosis and treatment.

**Methods:**

Four patients were found to have NEN primaries metastasised to the breast. A literature review was conducted to identify similar cases and characterise breast metastases from neuroendocrinal tumors (NETs).

**Results:**

Two patients presented with bilateral breast metastases (one with well-differentiated panNET and another with atypical lung carcinoid) and two had unilateral (one with moderately differentiated lung NET and one with atypical lung carcinoid). There are about 13 cases of NEN breast metastases reported in the English literature. The ileum is the most common primary site, followed by the appendix, duodenum, pancreas and lung.

**Conclusion:**

Breast lesions from extramammary primary often pose a diagnostic challenge, since a breast nodule can be the first and often the only presentation of the disease. However, differentiating between primary and secondary NEN breast lesions is essential, owing to different clinical management and prognosis.

## Introduction

Breast metastases from extramammary malignancies are uncommon, representing approximately 2% of breast masses [1, 2]. The contralateral breast, haematological malignancies, melanomas, carcinomas and sarcomas are the primary sites for the majority of breast metastases [3–6]. Most cases have been reported in female patients, while male breast metastases seem to be very infrequent [7–10]. Bilateral metastases to the breast are even more uncommon, with only very few reported in the literature [11]. Primary neuroendocrine neoplasms (NENs) in the breast are very rare. Up until 2011, the prevalence was less than 0.1% of all breast lesions and less than 1% of all NENs, whereas metastatic NENs lesions involving the breast represent only 1%–2% of all breast tumours [1]. However, it seems that over the last 5 years the diagnostic frequency of primary breast NENs has increased, leading to an estimated prevalence of 2%–5% of diagnosed breast cancers, affecting mostly the elderly population aged between 60 and 80 years [12–16]. The first case of a NEN metastasis excised from the breast of a 72-year-old woman was described by Chodoff [17]. Although recent data suggests that breast secondaries from NENs maybe more frequent than what was thought, based on the idea that many of them have been misdiagnosed in the past as primary NENs or carcinomas [4, 18, 19], they remain a rare phenomenon with no more than 200 cases published in the English literature to date [4, 18–48].

**Figure 1.**
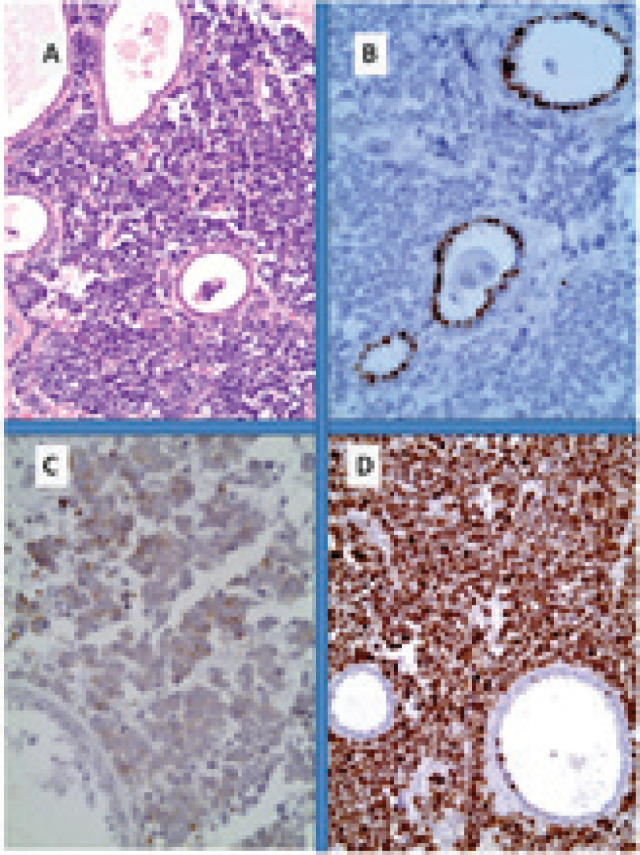
Primary small cell neuroendocrine carcinoma of the breast. (A): Neuroendocrine tumour infiltration from sheets of uniform cells with round nuclei; (B): immunohistochemistry for oestrogen receptor shows nuclear reactivity only in non-neoplastic ductal cells while tumour cells are negative; (C): at least for 50% of the population it shows cytoplasmic reactivity for chromogranin and (D): Very high Ki67 proliferative index.

Breast lesions from extramammary primary often pose a diagnostic challenge, since a single breast nodule can be the first and often the only presentation of the disease [48]. In fact, the literature reveals cases of NENs metastatic to the breast with an occult primary carcinoid tumour. However, differentiating between primary and secondary breast lesions is essential, owing to different clinical management and prognosis [41, 48] (Figure 1). The ileum is the most common primary site of metastatic NEN breast lesions, followed by the appendix, duodenum, pancreas, lung and ovary [5, 33, 42, 43]. With regard to cases of bilateral breast metastases from a distant NEN primary, there are about 13 cases reported in the English literature [4, 25, 31, 32, 43, 49–53]. The first case of bilateral breast metastases was from a duodenal carcinoid, published by Hawley [54].

## Method

The cases described in our article were identified and treated at the European Institute of Oncology of Milan, Italy. A literature review was conducted to identify similar cases and characterise features of bilateral or unilateral breast metastases from neuroendocrine tumours.

## Results

### Case 1: Bilateral breast metastases from a well-differentiated pancreatic neuroendocrine tumour

**Figure 2.**
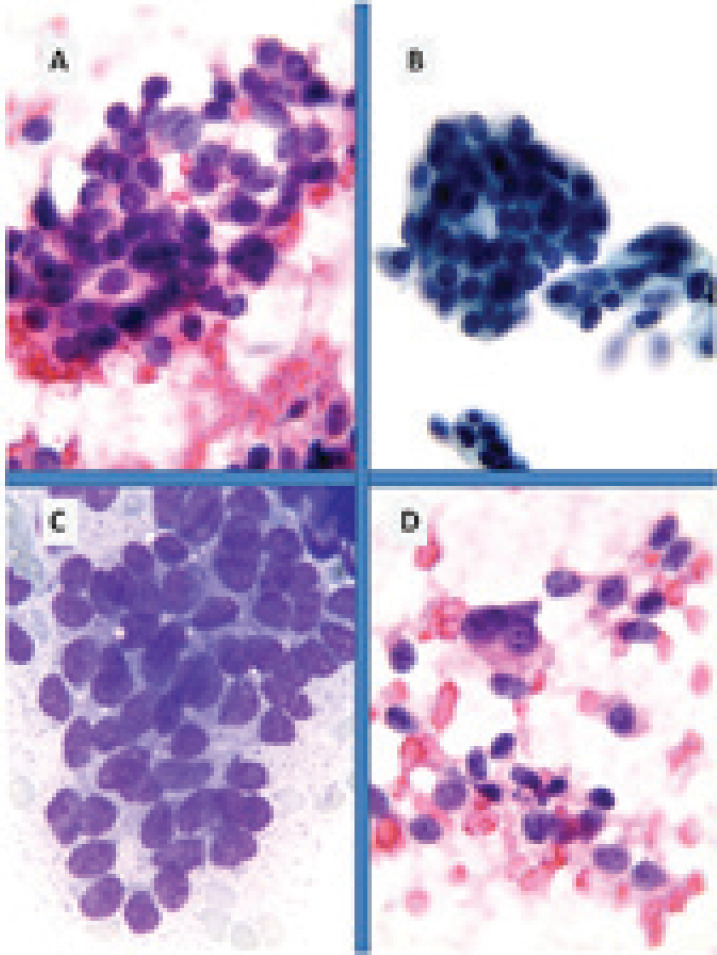
Breast metastases from pancreatic NET (FNA): Cytopathology could not give information about tumour biological features; the neoplastic elements are uniform and consist of small cells with scant cytoplasm, salt-and-pepper chromatin and micronucleoli and consistent neuroendocrine tumour (A and D: HE 40×; B: Papanicolau 40×; C: Giemsa 100×).

**Figure 3.**
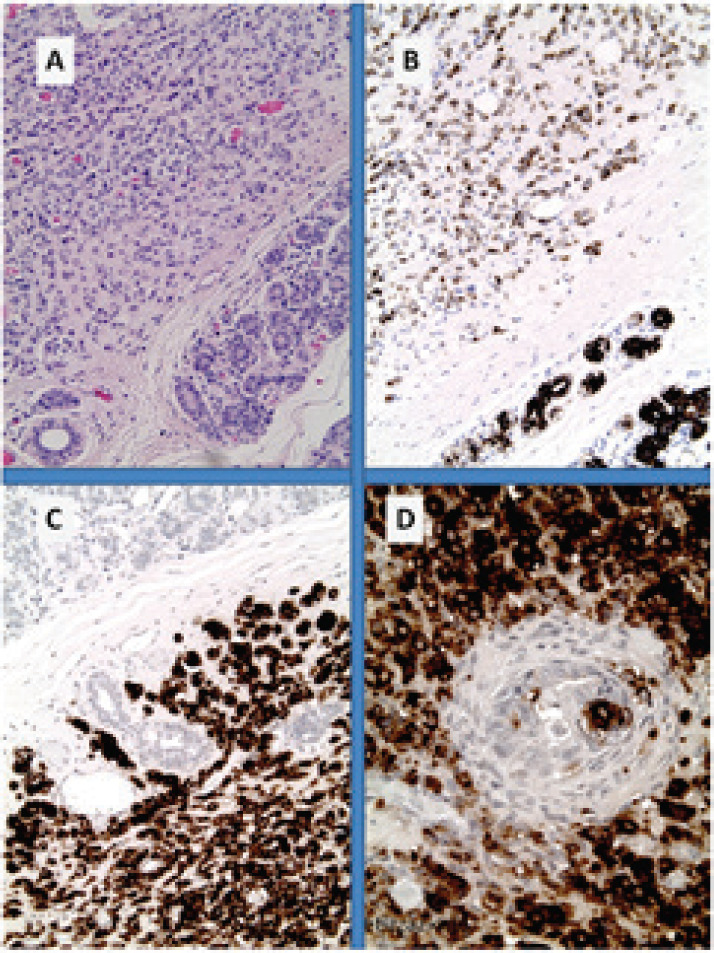
Breast metastases from pancreatic NET (inset: core biopsy). (A): nests of uniform cells with round nuclei; (B): with high proliferative index of 18% by Ki67; (C): immunohistochemistry for chromogranin A shows diffuse cytoplasmic reactivity in tumour cells and (D) on the other hand, only the lobular structures show positivity for CK7.

The first case is that of a 40-year-old woman with a family history of gastric cancer, who presented at our centre after having a pancreaticoduodenectomy for a pancreatic tumour with liver metastases in April 2006. Histology of the primary and biopsy of the liver had shown a well-differentiated endocrine carcinoma (WHO classification 2000), but no other information on Ki67 or other biological features were available. Post-operative somatostatin receptor scintigraphy (SRS) was negative. She was started on somatostatin analogue 30 mg/die (SSA) for 3 months, when the hepatic lesions showed evidence of progression of disease (PD) in October 2006. She was then started on Thalidomide (100 mg/day) and Temozolomide (150 mg/die for 7 days) chemotherapy, followed by loco-regional treatment of liver metastases with transarterial embolisation (TAE) and trans-arterial chemoembolisation (TACE) until February 2008. Despite an initial response, she had a slowly but steadily progressive disease on computerised tomography (CT) scan and a rising chromogranin A (CgA) level. In March 2008, a new lesion appeared on the external upper quadrant of the right breast on mammogram. The ultrasound scan confirmed a mass of 10-mm diameter. Cytological examination of the breast nodule initially suggested a breast carcinoma. However, considering the background of the existing metastatic NEN disease, fine needle aspiration (FNA) was repeated but was inconclusive, showing malignant tumour cells (Figure 2). A tru-cut biopsy was carried out which finally confirmed our suspicion of a metastasis from the well-differentiated endocrine carcinoma with a mitotic cell count (Ki67) of 18%, positive for CgA and synaptophysin (SYN), negative for CDX-2, ER/PgR and Her-2 (Figure 3). In July 2008, she was started on capecitabine (2,000 mg/die) and the disease remained stable till May 2009, when the magnetic resonance imaging (MRI) showed an increase of the right breast lesion to 17-mm diameter and the appearance of smaller nodules bilaterally, the largest being 1-cm diameter. The patient was started on Everolimus 10 mg/die, which was stopped after few days due to symptoms of cystitis related to treatment. After this therapy, her general physical condition gradually declined. She developed persistent diarrhoea as sign of disease and became severely dehydrated, leading to a hospital admission. After her hospitalisation, she was started on Octreotide long-acting release (LAR). The patient was last seen in the outpatient clinic in November 2009 with a follow-up CT scan showing extensive disease with multiple metastases in the liver, lungs, breast, bones and lymph nodes. The patient died in December 2009 due to PD.

### Case 2: Bilateral breast metastases from an atypical lung carcinoid

The second case we report is that of a 60-year-old female with a previous history of smoking (10 pack-years). She underwent a chest X-ray due to persistent cough, followed by a CT scan of the thorax. A 3.5-cm mass along with multiple smaller satellite nodules (max diameter 1 cm) were seen on the right hemithorax. A fine needle aspiration biopsy (FNAB) resulted in a poorly differentiated carcinoma. In September 2005, she underwent a right pneumonectomy and a lymphadenectomy with histological diagnosis of atypical carcinoid (AC) [55]. The stage was pT4pN1 according to the TNM staging system and the tumour features were CgA and SYN positive, TTF-1 negative, mitotic cell count (Ki67) of 5%, which were confirmed by two pathologists with definitive diagnosis of atypical lung carcinoid. The postoperative CT scans of chest and abdomen were negative. Radiological and clinical follow-up did not show any recurrence of disease until January 2006, when an SRS and a [18F]-2-fluoro-2-deoxy-d-glucose positron emission tomography (FDG-PET) scan showed high uptake on the breasts. Mammogram confirmed the presence of at least four bilateral breast nodules, the largest was located in the upper external quadrant of the right breast (1.8 cm) and in the lower periareolar area of the left breast (1.5 cm). Histological examination of both the nodules was diagnostic of neuroendocrine tumour, with CgA positive, SYN negative, ER/PgR negative and Ki67 of 15%. In March 2006, the patient underwent a bilateral breast lumpectomy and a sentinel lymph node biopsy. The histological diagnosis was consistent with breast metastases from pulmonary carcinoid grade 2 (WHO 2010) with Ki67 of 15%, CgA, SYN and TTF-1 positive, ER/PgR negative and HER-2 negative. She received adjuvant chemotherapy with six cycles of carboplatin and etoposide with a good response. In November 2006, the patient started developing focal neurological signs with right eyelid ptosis and an MRI of the brain showed metastatic lesions. She received whole brain radiotherapy (3,250 cGy) in September 2007. The disease remained fairly stable up until February 2008 when new bilateral multiple breast lesions appeared on mammogram, along with brain metastases and pathological mediastinal lymph nodes (LFNs) and lesions on whole body CT scan and FDG-PET scan. In May 2008, peptide receptor radionuclide therapy (PRRT) was started up to December 2008 (with total dosage of 240 mCi of 177Lu-DOTATE) when a CT scan showed PD in the breast, lung and LFNs. The patient was scheduled for 68Ga PET/CT, which she failed to attend, and was lost from follow-up since.

### Case 3: Unilateral breast metastasis from a moderately differentiated lung neuroendocrine tumour

**Figure 4.**
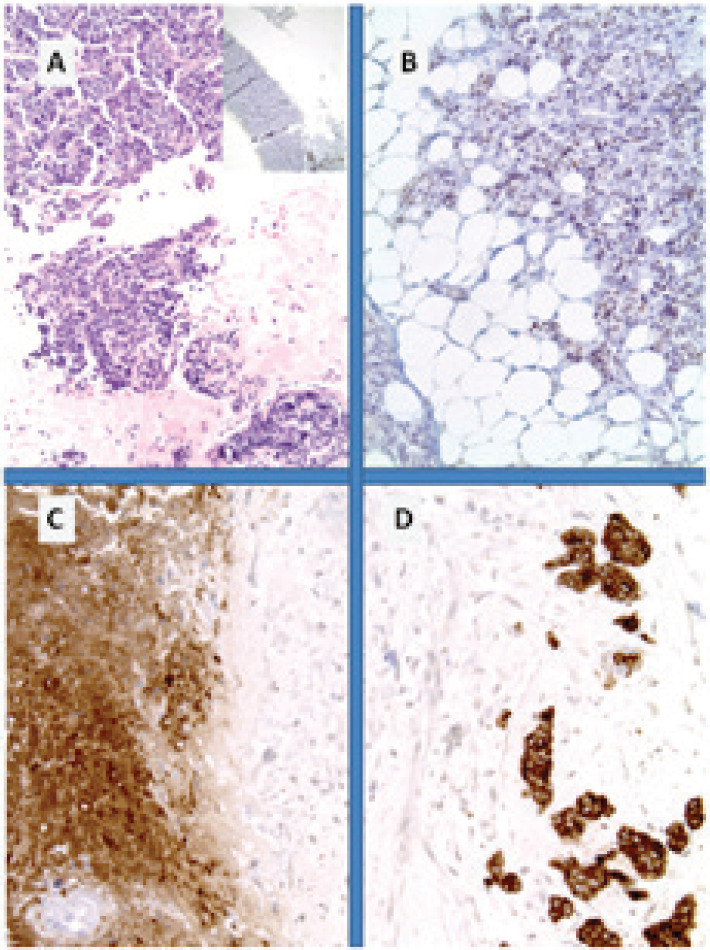
Metastatic breast NET from lung carcinoid. (A): Breast tissue infiltrated in lobular carcinoma fashion; (B): immunohistochemistry for CK7 and for synaptophysin; (C): diffuse cytoplasmic reactivity in tumour cells and (D) with typical pagetoid involvement of the hyperplastic duct.

This is the case of a 50-year-old female, with a family history of pancreatic cancer, who was found to have a chest lesion on chest X-ray carried out in 2006 because of intermittent cough since 2000. A subsequent CT scan of the thorax confirmed a 13-mm nodule on the upper right lobe of the lung, which did not seem suspicious for malignancy. Following a FDG-PET, which did not pick up on the nodule (SUV max 1.25), observation of the lesion was decided with yearly scans. Up until 5 years later, the lesion seemed to have remained unchanged. However, the imaging follow-up of December 2010 showed a new lesion in the right breast. In January 2011, she underwent right breast quadrantectomy for a lump histologically perceived as an infiltrating triple negative lobular carcinoma, with a negative sentinel node. This result was confirmed from a second pathologist later on. Surgery was followed by adjuvant chemotherapy and radiotherapy, completed in July 2011. In August 2012, a left breast nodule was detected on mammogram and confirmed on MRI. Biopsy of the lesion resulted in a triple negative invasive ductal carcinoma. A PET/CT with FDG was carried out which surprisingly showed a high uptake only on the old pulmonary nodule, which had increased in the meantime, becoming 3 cm in diameter (SUV max 5.9). Biopsy of the lesion was suspicious for a carcinoid. In June 2012, the patient underwent a right lobectomy and right hilomediastinic lymph node dissection. Histology reported a neuroendocrine neoplasm with Ki67 of 18%. Clinically, the patient showed PD from the left breast with subcutaneous nodules formation. It was decided to proceed to a bilateral mastectomy with subcutaneous nodules removal in July 2012. The histological result showed a neuroendocrine tumour, Ki67 of 12% and negative left sentinel node. A second pathology opinion was concluded for breast metastases and subcutaneous nodules from a moderately differentiated NEN of pulmonary origin, with Ki67 of 14% positive for CgA and TTF-1 and negative for calcitonin and somatostatin receptors (SSTRA2), concluding for an AC (Figure 4). A follow-up 68Ga-PET/DOTATOC, CT with contrast medium and MRI showed further lesions on left and right femur. Biopsy of the left femoral lesion confirmed a metastasis from the same moderately differentiated NEN with Ki67 of 16% with negative somatostatin receptor (SSTR). In view of disease progression, it was decided to start chemotherapy in January 2013 with capecitabine (1,500 mg/m^2^/die for 14 days) and temozolomide (150 mg/m2 for 5 days). The patient tolerated well the first six cycles which were stopped due to thrombopenia (grade 3) and malaise and were switched to subcutaneous (SC) octreotide LAR 30 mg/die. A follow-up CT scan showed a new pulmonary nodule of 3 mm on the left and an ultrasound of the thyroid revealed nodule on the right lobe of 15 × 12 mm, which was aspirated and confirmed the metastatic nature of the primary pulmonary lesion. The patient was reluctant to undergo for further chemotherapy at that time, and thus decided to continue with the somatostatin analogue and re-evaluate the situation in few months. In August 2013, her disease was clinically and radiologically stable.

### Case 4: Unilateral breast metastasis from an atypical lung carcinoid

This is the case of a 68-year-old woman with a past medical history of a right radical mastectomy for a primary breast carcinoma at the age of 40. In 2004, she was admitted to the hospital for a suspected pulmonary embolism. The CT scan of the thorax revealed a 2-cm mass occluding the left bronchus. In October 2004, she underwent left inferior lobectomy and was histologically diagnosed as having an atypical pulmonary carcinoid. The follow-up was negative until October 2007, when a nodule in the right lobe of the thyroid was detected. FNAB revealed a suspected papillary neoplasm and she underwent total thyroidectomy in January 2008. The histological analysis indicated a metastasis from the lung NEN. Three months post-thyroidectomy, CT of chest/abdomen, SRS and 68Ga PET scans were all negative. However, FDG-PET revealed a 7-mm nodule in the left breast; thus, she underwent quadrantectomy in September 2008. The histological diagnosis was a NEN metastasis that was positive for CgA, SYN and CD56, and negative for ER, PGR, HER-2 and negative for SSTR 2, 3 and 5. In January 2009, she was restaged with a chest X-ray, thyroid ultrasound and bone scan, Blood CgA and neuron-specific enolase (NSE), as well as imaging was negative for PD.

## Discussion

NENs are a heterogeneous group of uncommon malignancies originating from the diffuse endocrine system. While poorly differentiated NENs have an aggressive behaviour with a poor prognosis, well-differentiated NENs are usually slowly progressing even though they can give metastatic spread to distant sites, mainly to the liver. In 2010, the WHO published the new classification of gastroenteropancreatic (GEP) NENs based on proliferation index (Ki-67) and/or mitotic index (MI), aiming to differentiate between tumours [neuroendocrinal tumors (NETs)] and carcinomas [neuroendocrinal carcinomas (NECs)]. In particular, NETs include grade 1 (Ki67 < 2% and/or MI ≤ 2/10 high power field, HPF) and grade 2 (3% < Ki67 < 20% and/or 3 < MI < 20/HPF) NENs, whereas NECs refer to the grade 3 (Ki67 > 20% and/or MI > 20/HPF) [56]. This grading is a strong predictor of prognosis. In fact, in the well to moderately differentiated NETs, survival at 5 years reaches 35%, whereas in the poorly differentiated NECs it is less than 5% [57]. On the other hand, according to the 2004 WHO classification, four major types of lung neuroendocrine neoplasms are recognised: typical carcinoid, AC, small cell lung cancer (SCLC) and large cell neuroendocrine carcinoma [55].

NENs can develop in any part of the body, most commonly in the gastrointestinal tract. The ileum was found to be the most common primary site of metastatic breast NENs. The appendix, duodenum, pancreas, lungs and ovaries were the other primary sites from where NENs metastasise to the breast [5, 33, 36, 42, 43]. Breast metastases from NENs are not common, although there is an increased frequency reported over the last years. This could be the result of more NEN breast metastases being correctly diagnosed, as specialists are more conscious that these lesions can resemble primary carcinomas and modern technology imaging aids in reaching the correct diagnosis [5, 18, 19, 51]. In particular, an increased occurrence of breast metastases from GEP NENs has been reported [58]. One of the first cases of metastatic NEN to the breast was published in 1977. This was the case of a 58-year-old woman who presented with an isolated breast mass, which was subsequently found to be the metastasis from a bronchial carcinoid [27, 59]. In fact, a solitary breast lump may often be the first and only manifestation of the disease and can mimic a primary breast carcinoma [25, 48, 51]. The mean age of presentation for metastatic breast NENs is considered to be 56 years, which is by 10 years younger than the patients presenting with primary NENs of the breast, usually in their sixth and seventh decade of life [22]. However, the presentation of bilateral NEN metastases to the breast remains an extremely rare manifestation of the disease [1, 31, 32, 60].

Breast metastases from NEN represent an important diagnostic challenge for practitioners because of the difficulty to differentiate from a primary breast carcinoma. An accurate study of the tumour characteristics and patient clinical history are necessary in order to decide on adequate medical and surgical treatment [32, 36, 41, 43, 48]. Histology and immunohistochemistry can be useful in the recognition of the neuroendocrine structure of the tumour. Neuroendocrine tumours typically form nests or sheets of uniform cell populations with abundant eosinophilic cytoplasm and round nuclei. At least 50% of the cell population must be immunoreactive for at least one neuroendocrine marker, including SYN, CgA and B, NSE and negative for cytokeratin 7, whereas breast carcinoma strongly express cytokeratin 7. The SSTR expression is usually negative in breast carcinoma unlike NENs (even though in some cases NENs may lack expression of SSTR expression). Oestrogen and progesterone hormone receptors do not help to differentiate breast carcinoma from primary breast NEN, as may be positive in both cases, whereas metastatic NENs are typically negative for hormone receptors and Her-2 [1, 20, 29, 34, 61]. In fact, immunohistochemical determinations in all the cases we reported showed a positive staining for chromogranin A and synaptophysin, while ER, PgR and HER-2 were not expressed, confirming the neuroendocrine nature of the lesions. In two of the above-mentioned cases, SSTR immunohistochemical determination was carried out and the absence of somatostatin receptors could explain why the SRS and 68Ga PET did not pick up any abnormality. Nevertheless, FNA is considered to be the best diagnostic procedure for a correct diagnosis of breast nodules [30, 32, 62, 63]. It is worth mentioning that this needs to be carried out with caution, as it can precipitate a carcinoid crisis in the case of hormonally active tumours [20]. In addition, it has been shown that histological analysis and morphological evaluation with the use of auxiliary immunohistochemical studies are important in reaching a correct diagnosis, particularly in difficult cases such as those with an unknown primary [32, 36]. Morphological distinction is often difficult on both FNA and lumpectomy specimens due to several overlapping features and the reported frequency of neuroendocrine cells (–25%) in breast carcinoma [13, 64–68].

Moreover, morphological and functional imaging can play an important role in differential diagnosis. At mammographic evaluation, the breast NET appears as a more round circumscribed mass, while the breast carcinoma has more speculated edges and often has evidence of microcalcifications, which are usually absent in a NEN of the breast [67, 69].

On the other hand, radiological imaging cannot offer much in differentiating the various lesions. The appearance of breast nodules on MRI and ultrasound is substantially similar to the case of metastatic and primary NENs, as well as the case of breast carcinoma [22, 26, 69]. Additional radionuclide imaging can be useful in differentiating NENs, as the majority of them express SSTR compared to ductal mammary carcinomas [25, 28, 70]. A recent study showed that 68Ga PET/CT-DOTATOC in patients with NEN enables detection of cardiac and breast metastases from NENs, as well as other rare sites [19]. However, imaging characteristics alone are not sufficient for a definitive diagnosis. Fine needle aspiration or core needle biopsy is necessary to confirm or exclude suspicion. Finally, also an in-depth medical history may play an important role in differentiating a primary from a breast metastasis. Previous history of NENs, even if radically treated or with low-grade features, needs to be considered as a potential primary of future metastases. To a lesser extent, synchronous or subsequent second primary malignancies related to NEN have also been reported [46].

There are no clear recommendations about the surgical approach of these tumours. It is suggested that a localised primary breast NEN should be treated as an invasive ductal carcinoma with mastectomy or breast conserving therapy with lumpectomy and negative margins, as well as axillary staging with sentinel lymph node biopsy [1, 71–73]. However, confirming negative surgical margins can be challenging, as neuroendocrine carcinomas may have pagetoid involvement or a background of hyperplasia can produce artefacts [74, 75]. With regard to patients with breast metastases from NEN, a lumpectomy alone is recommended, whereas mastectomy is advisable only if there are numerous large metastatic NENs to the breast. Multiple resections would be recommended in the presence of more than one lesion, aiming to locally control the disease and preserve the breast [41, 76]. Debulking of metastases often offer better survival compared to no resection [77]. If palpable adenophathy is absent, axillary lymph node dissection is not deemed necessary [41]. Accurate recognition of the entity is vital to avoid unnecessary treatment [25].

In addition to surgical treatments, the need for medical therapy either locally or more frequently systemic arises, depending on the site and extension of metastases, clinical history and symptoms. In case of unresectable and symptomatic NEN hepatic metastases, transarterial liver-directed therapies, such as radiofrequency thermal ablation, TAE, TACE or selective internal radiation therapy could be an option for symptomatic relief and reduction of hormone levels [78]. Systemic treatments may consist of fluoropyrimidine, oxaliplatin or temozolomide-based chemotherapy, depending on previous treatments and the primary tumour site. If SSTR expression on SRS or 68Ga PET scan is present, PRRT treatment may be indicated to control tumour growth, combined with SSAs for symptoms control, especially in case of carcinoid syndrome [79, 80].

We report the cases of four female patients who developed breast lumps, two bilateral and two unilateral, mimicking the clinical and histological features of breast carcinomas on a background of previous NENs; two atypical lung carcinoids, a well-differentiated pancreatic NET and a moderately differentiated NET from pulmonary origin. We observed that in the majority of the reported cases, patients were initially misdiagnosed, mainly as having mammary carcinomas, resulting in inappropriate breast surgery and axillary dissection instead of systemic specific treatment. Only later the histological review of surgical specimens led to the correct diagnosis, as it happened in our last case. However, suspicion may not always be raised, particularly in the cases where the primary tumour was never found [4, 71]. Patients presenting with breast nodules and having a past history of NENs or typical symptoms of carcinoid syndrome (flushing, watery diarrhoea, abdominal pain and tachycardia) must always be considered as having a potentially metastatic disease, even in the case of a previous well-differentiated NET. Since it is not always possible to detect a primary extramammary NET, a single breast lesion can be easily thought as the primary NET. Nonetheless, this term is subject to controversies and it is thought that a ‘mammary carcinoma with endocrine features’ is more adequate [19, 26, 28, 62, 63]. Clinically, there are no criteria to distinguish metastatic breast NET from primary breast endocrine tumours [69]. *In situ* ductal carcinoma with neuroendocrine expression may be the only proof of the primary nature of the breast lesion. Although a challenge, meticulous investigations and medical history are crucial in order to reach the correct diagnosis and differentiate between primary and secondary breast tumours.

## Conclusion

Breast metastases from NETs are not seen often in clinical practice; nevertheless, practitioners need to be alert and investigate in detail a patient presenting with a breast mass in order to rule out metastasis. Bilateral breast metastases originating from NETs are exceptionally rare. However, it is important to differentiate between a primary and secondary breast lesions, as this will define management and prognosis. Clinical and histological similarities make diagnosis particularly difficult at times, especially when there is an absence of a known primary. In addition, clear guidelines regarding management are lacking and yet more experience is needed to determine the best approach. A practitioner should consider all aspects when deciding a treatment option. Tumour features, patient’s clinical status and perspectives, as well as short and long-term objectives will all need to be taken into account in order to choose the best therapeutic strategy.

## Conflicts of interest

The authors have nothing to disclose.

## Funding

This research did not receive any specific grant from any funding agency in the public, commercial or not-for-profit sector.
